# Application of pattern recognition techniques for classification of pediatric brain tumors by in vivo 3T ^1^H‐MR spectroscopy—A multi‐center study

**DOI:** 10.1002/mrm.26837

**Published:** 2017-08-08

**Authors:** Niloufar Zarinabad, Laurence J. Abernethy, Shivaram Avula, Nigel P. Davies, Daniel Rodriguez Gutierrez, Tim Jaspan, Lesley MacPherson, Dipayan Mitra, Heather E.L. Rose, Martin Wilson, Paul S. Morgan, Simon Bailey, Barry Pizer, Theodoros N. Arvanitis, Richard G. Grundy, Dorothee P. Auer, Andrew Peet

**Affiliations:** ^1^ Institute of Cancer and Genomics Sciences University of Birmingham Birmingham United Kingdom; ^2^ Birmingham Children's Hospital Birmingham United Kingdom; ^3^ Department of Radiology Alder Hey Children's NHS Foundation Trust Liverpool United Kingdom; ^4^ Department of Imaging and Medical Physics University Hospitals Birmingham NHS Foundation Trust Birmingham United Kingdom; ^5^ The Children's Brain Tumour Research Centre University of Nottingham Nottingham United Kingdom; ^6^ Medical Physics, Nottingham University Hospital, Queen's Medical Centre Nottingham United Kingdom; ^7^ Neuroradiology, Nottingham University Hospital, Queen's Medical Centre Nottingham United Kingdom; ^8^ Neuroradiology Department Newcastle upon Tyne Hospitals Newcastle upon Tyne United Kingdom; ^9^ Centre for Human Brain Health, School of Psychology, University of Birmingham Birmingham United Kingdom; ^10^ Radiological Sciences, Department of Clinical Neuroscience University of Nottingham Nottingham United Kingdom; ^11^ Paediatric Oncology Department Great North Children's Hospital Newcastle upon Tyne United Kingdom; ^12^ Department of Paediatric Oncology Alder Hey Children's NHS Foundation Trust Liverpool United Kingdom; ^13^ Institute of Digital Healthcare WMG, University of Warwick Coventry United Kingdom

**Keywords:** MR spectroscopy, 3T, pediatric brain tumors, diagnosis, classification

## Abstract

**Purpose:**

3T magnetic resonance scanners have boosted clinical application of ^1^H‐MR spectroscopy (MRS) by offering an improved signal‐to‐noise ratio and increased spectral resolution, thereby identifying more metabolites and extending the range of metabolic information. Spectroscopic data from clinical 1.5T MR scanners has been shown to discriminate between pediatric brain tumors by applying machine learning techniques to further aid diagnosis. The purpose of this multi‐center study was to investigate the discriminative potential of metabolite profiles obtained from 3T scanners in classifying pediatric brain tumors.

**Methods:**

A total of 41 pediatric patients with brain tumors (17 medulloblastomas, 20 pilocytic astrocytomas, and 4 ependymomas) were scanned across four different hospitals. Raw spectroscopy data were processed using TARQUIN. Borderline synthetic minority oversampling technique was used to correct for the data skewness. Different classifiers were trained using linear discriminative analysis, support vector machine, and random forest techniques.

**Results:**

Support vector machine had the highest balanced accuracy for discriminating the three tumor types. The balanced accuracy achieved was higher than the balanced accuracy previously reported for similar multi‐center dataset from 1.5T magnets with echo time 20 to 32 ms alone.

**Conclusion:**

This study showed that 3T MRS can detect key differences in metabolite profiles for the main types of childhood tumors. Magn Reson Med 79:2359–2366, 2018. © 2017 The Authors Magnetic Resonance in Medicine published by Wiley Periodicals, Inc. on behalf of International Society for Magnetic Resonance in Medicine. This is an open access article under the terms of the Creative Commons Attribution License, which permits use, distribution and reproduction in any medium, provided the original work is properly cited.

## INTRODUCTION

Brain tumors are a significant cause of death and long‐term disability in children, with a range of treatment options and outcomes depending on the tumor type, location, and age of the patient. Histopathology following biopsy or surgical resection is the current gold standard for diagnosis of brain tumor type and grade 1. Although surgical resection is often appropriate for many tumors and also provides a histological diagnosis, there are distinct advantages to having a diagnosis before surgery. A pre‐operative diagnosis can influence the extent of surgical resection attempted, allow the timely planning of adjuvant treatment and aid discussions with the family. Conventional magnetic resonance imaging (MRI) is commonly used to propose a diagnosis before surgery but is of limited accuracy. A previous study reviewed the radiological reports from a cohort of children with medulloblastoma, pilocytic astrocytoma, or ependymoma and showed an accuracy of diagnosis of 66% 2.

MRI techniques have significantly advanced in recent years with new imaging techniques being able to provide information on tissue properties, structure, and basic metabolic processes. Amongst these new techniques, ^1^H‐MR spectroscopy (MRS) has the ability to provide non‐invasive measurements of metabolite profiles with the potential to aid diagnosis and improve the characterization of pediatric brain tumors 3, 4, 5, 6, 7, 8.

Spectroscopic studies on pediatric brain tumors have attempted to characterize different histologic types and predict the degree of malignancy 7, 9. Non‐invasive grading is especially important for tumors in the eloquent areas or deeply located tumors. Single and multi‐center studies have worked on development and optimization of diagnostic classifiers of childhood brain tumors using MRS. This has yielded promising results in discriminating between certain common tumor types 6, 7.

With the integration of 3T MRI into clinical practice, there is growing interest in the practical improvement of MRS at a 3T field strength over the more established magnetic field strength of 1.5T, because both the spectral resolution and the spatial resolution depend, in a linear fashion, on the magnetic field 10, 11, 12.

Although single‐voxel MRS of the human brain has been carried out at many field strengths (from 0.5T to 7T), to date, no study has been reported on application of pattern recognition techniques for classification of pediatric brain tumors using 3T MRS.

The aim of this study was to evaluate the discriminative potential of metabolites obtained from 3T MRI scanners in classifying pediatric brain tumors by comparing the performance of three different pattern recognition techniques.

## METHODS

### Patients

This study included 52 patients less than 16 years of age (8.2 ± 5.32 years, 21 female and 31 male) with histologically proven brain tumor collected from four centers in the United Kingdom. Patient data were collected retrospectively from children who underwent single‐voxel MRS between November 2009 and April 2016 during a routine MRI for a suspected brain tumor before treatment.

The enrolled cohort consisted of patients with three different tumor types from all regions of the brain, including medulloblastoma (MB) (*n* = 18), pilocytic astrocytoma (PA) (*n* = 26), and ependymoma (EP) (*n* = 8). Histopathological, clinical, and radiological features were used to form a diagnosis agreed by a multidisciplinary team. Approval was obtained from the research ethics committee and informed consent given by parents/guardians.

### Data Acquisition

All studies were performed using 3T scanners from different manufacturers (Philips Achieva, Siemens MAGNETOM Verio). MRS was performed after conventional MRI that included T_1_‐ and T_2_‐weighted and T_1_‐weighted post‐contrast sequences. Spectroscopy images were acquired using a point resolved single voxel spectroscopy (PRESS) sequence (time echo = 30–46 ms, pulse repetition time = 2000 ms). Cubic voxels size varied from 3.38 cm^3^ to 8 cm^3^ and 128 repetitions were used. A water unsuppressed acquisition was also acquired as a concentration reference. Conventional MRI was used for guiding voxel placement to ensure it is entirely located within the tumor

### MRS Processing

Raw spectroscopy data were processed using TARQUIN (version 4.3.6) 13 fitting to a linear combination of 19 metabolite basis functions generated at the correct field strength and echo time with an additional 9 lipid and macromolecular components 13, 14, 15. Frequency alignment, zero order phase correction, baseline correction, and water removal using Hankel singular value decomposition (HSVD) methods were applied by TARQUIN. TARQUIN determines the chemical shift offset, phase, and baseline during the fitting process. It then zero fills the time domain data by a factor of 2 (×2) and converts the time domain signal to spectral domain using a Fourier transform. The obtained spectra are then resampled to 0.49 Hz/point. This is to ensure all cases have a consistent Hz/point. The resampled spectra are used for analysis in this study. The full spectral range (−3.00 to 12.5 ppm) was used for fitting the data and metabolite quantitation. Quantitation was carried out relative to a water reference spectrum. Corrections for T_2_ relaxation times of metabolites and water were applied as the default values for TARQUIN 16. To include the metabolites of interest in the study, main metabolites in 0.5 to 4 ppm region were used for classification.

Out of 52 enrolled cases, 41 cases (medulloblastoma *n* = 17, pilocytic astrocytoma *n* = 20, and ependymoma *n* = 4, of which 1 is analplastic ependymoma) passed the following quality control criteria: signal‐to‐noise ratio obtained from TARQUIN (SNR)≥4 (here SNR is defined as ratio between the maximum in the spectrum minus baseline divided by 2 × root‐mean‐square of the spectral noise level), full‐width half‐maximum obtained from TARQUIN (FWHM) ≤ 0.15 ppm, stable baseline, good phasing, adequate water suppression, and absence of artefacts. The voxel position was also reviewed to ensure it was positioned over tumor, did not include significant amounts of normal‐appearing brain or cyst, and was at least 3 mm away from lipid‐containing bone and scalp. The 11 failed cases include medulloblastoma *n* = 1, pilocytic astrocytoma *n* = 6, and ependymoma *n* = 4. A poor voxel placement that included normal brain, small voxel size, and low quality spectra were the main quality control failure reasons for the latter cases.

For the quantified metabolite profiles, Crammer‐Rao lower bounds were calculated to evaluate metabolite accuracy. All metabolites where at least two patients had a Crammer‐Rao lower bound < 50 were included (alanine, aspartate and GABA were excluded) 7. The following metabolites were used for classification: citrate (Cit), glucose (Glc), glutamine (Gln), glutathione (Glth), glutamate (Glu), glycine (Gly), myo‐inositol (mIns), lactate (Lac), total choline (tCho = glycerophosphocholine [GPC] + phosphocholine [PCh]), scyllo‐inositol (scyllo), taurine (Tau), total creatine (tCr = Cr + PCr), and total NAA (tNAA = NAA + NAAG). The nine macromolecular and lipid components were grouped together to account for three broad resonances at 0.9 ppm (tLM09), 1.3 ppm (tLM13), and 2.0 ppm (tLM20), giving a maximum of 16 variables.

The Kruskal‐Wallis test for the analysis of variance (α = 0.05) was applied to determine the significant differences in metabolite concentrations between the three groups. Mann‐Whitney U tests were carried out to compare mean metabolite values between individual pair of groups. Statistical analysis was carried out using SPSS statistics software (version 21.0).

### Classification

Borderline synthetic minority oversampling technique (bSMOTE) 17 was used to overpopulate the original ependymoma group by 100% and correct for the skewness and class imbalance in the original data. Oversampled ependymoma was added to the original data sets to create an overpopulated metabolite feature set. Principal component analysis was used to reduce dimension of this overpopulated set and extract features that best discriminate between the three tumor groups.

Classifiers were trained using linear discriminative analysis (LDA), support vector machine (SVM), and random forest (RF) approaches. These techniques have low, medium, and high model flexibility, respectively. A radial basis (Gaussian) function kernel was chosen for SVM. LDA was trained using diagonal linear learner where all classes have the same diagonal covariance matrix.

Developed classifiers using the bSMOTE‐overpopulated sets were tested using the original data and the validity of the classifier was checked by comparison with the known histological assignment. Ten‐fold cross‐validation was used to evaluate the learning algorithm performance. To evaluate classifiers trained using bSMOTE data, the original data set is partitioned into *z = 10* pools in which one pool is used for evaluation while the remaining *z* − 1 pools are added to the bSMOTE ependymoma cases, obtained from oversampling ependymoma cases in the remaining *z* − 1 pools, and used for training to produce mean error rates, where z ∈{1, 2, …, Z}. The pools were then randomly rotated and the subsampling and permutation tests were repeated until all pools had been evaluated exactly once. This ensured that there were no bSMOTE ependymoma cases in any of the test sets. The analysis have been repeated 100 times and results been averaged.

G‐mean and F‐measure metrics were used for more precise performance evaluation in class imbalance learning. The balanced accuracy rate (BAR) of the learning algorithm, calculated as the mean of the accuracies for the three tumor types, is also reported here as a performance measure metric. In this study, all learning algorithms were developed in python 2.7 (Python Software Foundation, Wilmington, DE) using Scikit‐learn (Version 0.16.1) and Orange (Version 2.6a2) libraries.

## RESULTS

**Figure 1:**
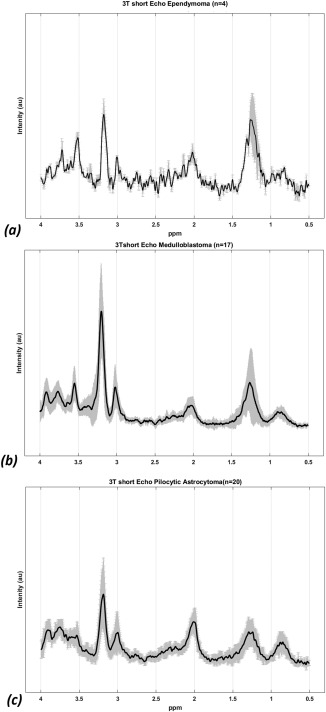
Mean 3T short time echo spectra for (**a**) ependymoma (*n* = 4), (**b**) medulloblastoma (*n* = 17), and (**c**) pilocytic astrocytoma (*n* = 20) with solid black line indicating the mean spectra and SD indicated by the shaded region.

Mean metabolite concentrations (mM) ± standard deviations (SD) for each diagnostic group are represented in Table 1. Analysis of variance revealed significant differences between ependymoma, pilocytic astrocytoma, and medulloblastoma in ten of the individual metabolites and combined macromolecules and lipids at 1.3 ppm. Glc, Gln, Glu, mIns, tLM09, and tLM20 had *P*‐values > 0.05. Results comparing metabolites between specific pairs of tumor types are reported in Table 2. MB demonstrated increased Tau and decreased mIns compared to EP. Compared with PA, MB had a number of significantly higher metabolites including tCho, Tau, scyllo, tCr, Gly, Glth, and tLM1.3. Compared with EP, PA demonstrated elevated tNAA and decreased tLM13 and mIns. Mean MRS spectra of the three tumor types is presented in Figure 1.

**Table 1:** Estimated Metabolite Concentration ± SD Calculated by TARQUIN^a^.

Metabolite	Ependymoma (*n* = 4)	Medulloblastoma (*n* = 17)	Pilocytic Astrocytoma (*n* = 20)	*P*‐Value
Cit*	0.96 ± 0.21	0.56 ± 0.31	0.47 ± 0.48	0.031
Glc	1.2 ± 0.27	2.3 ± 1.7	1.07 ± 1.06	0.073
Gln	1.69 ± 0.11	2.6 ± 1.53	2.2 ± 2.6	0.429
Glth*	1.12 ± 0.32	1.8 ± 0.8	0.66 ± 0.6	<0.000
Glu	1.14 ± 0.94	2.4 ± 2.2	3.2 ± 2.3	0.055
Gly*	1.92 ± 0.5	3.7 ± 2.08	0.73 ± 0.76	<0.000
mIns	4.53 ± 2.3	2.29 ± 1.66	1.94 ± 1.73	0.097
Lac*	1.39 ± 0.68	1.65 ± 1.7	0.65 ± 0.74	0.041
Scyllo*	0.008 ± 0.01	0.37 ± 0.47	0.05 ± 0.08	0.009
Tau*	0.6 ± 0.75	6.3 ± 4.1	1.07 ± 1.03	<0.000
tNAA* (tNAA = NAA + NAAG)	0.53 ± 0.25	1.03 ± 0.44	1.37 ± 0.95	0.007
tCho*	1.69 ± 0.29	3.94 ± 1.74	1.4 ± 0.709	<0.000
tCr*	1.69 ± 0.79	3.91 ± 1.5	2.29 ± 2.22	0.003
tLM09	3.7 ± 1.4	4.94 ± 2.4	3.02 ± 1.87	0.084
tLM13*	21.4 ± 10.05	19.32 ± 13.01	7.8 ± 3.7	<0.000
tLM20	7.2 ± 1.8	7.53 ± 4.1	6.3 ± 3.4	0.444

Cit, citrate; Glc, glucose; Gln, glutamine; Glth, glutathione; Glu, glutamate; Gly, glycine; Lac, lactate; mIns, myo‐inositol; NAA,N‐acetylaspartate; NAAG,N‐Acetylaspartylglutamic acid; scyllo, scyllo‐inositol; SD, standard deviation; Tau, taurine; tNAA, total N‐acetylaspartate; tCho, total choline; tCr, total creatine; tLM09, lipids and macromolecules 0.9; tLM13, lipids and macromolecules 1.3; tLM20, lipids and macromolecules 2.0.

a The *P*‐value of analysis of variance (calculated using Kruskal‐Wallis test with α = 0.05) is comparing ependymoma versus medullobastoma versus pilocytic astrocytoma.

*Metabolites with *P*‐values less than 0.05.

**Table 2:** Differentiation between Tumor Metabolite Profiles at 3T Comparing Those that Are Often Included in Differential Diagnoses on Conventional Radiology Using Mann‐Whitney U‐Test With *P*‐Values Reported.

	Ependymoma (*n* = 4)	Pilocytic Astrocytoma (*n* = 20)
Medulloblastoma (*n* = 17)	↓ Cit*	↑ Glc**
	↑ tCho**	↑ Glth***
	↑ tCr**	↑ Gly***
	↑ Tau**	↑ Lac*
	↓ mIns*	↑ Tau***
		↑ tCho**
		↑ tCr**
		↑ scyllo**
		↑ tLM09*
		↑ tLM13***

	Ependymoma (*n* = 4)	
Pilocytic astrocytoma (*n* = 20)	↓ Cit*	
	↑ Glu*	
	↓ Gly**	
	↑ tNAA**	
	↓ tML13*	
	↓ mIns*	

Cit, citrate; Glc, glucose; Glth, glutathione; Glu, glutamate; Gly, glycine; Lac, lactate; mIns, myo‐inositol; scyllo, scyllo‐inositol; Tau, taurine; tNAA, total N‐acetylaspartate; tCho, total choline; tCr, total creatine; tLM 09, lipids and macromolecules 0.9; tLM 13, lipids and macromolecules 1.3.

*P ≤ 0.05; **P < 0.01; ***P < 0.001.

**Figure 2:**
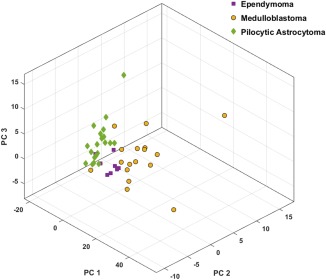
3D scatter plot presents principal component analysis (PCA) space for the discrimination of medulloblastoma (MB), pilocytic astrocytoma (PA), and ependymoma (EP).

Principal component analysis was carried out and principal components accounting for 95% of variance were extracted, giving four principal components. Figure 2 shows the three‐dimensional scatter plot of the three tumor groups using the first three principal components. Data points of each tumor types demonstrated a good degree of data clustering and separation. The four principal components were then submitted to SVM, RF, and LDA learning algorithms and classifiers were compared in view of their performance.

**Figure 3:**
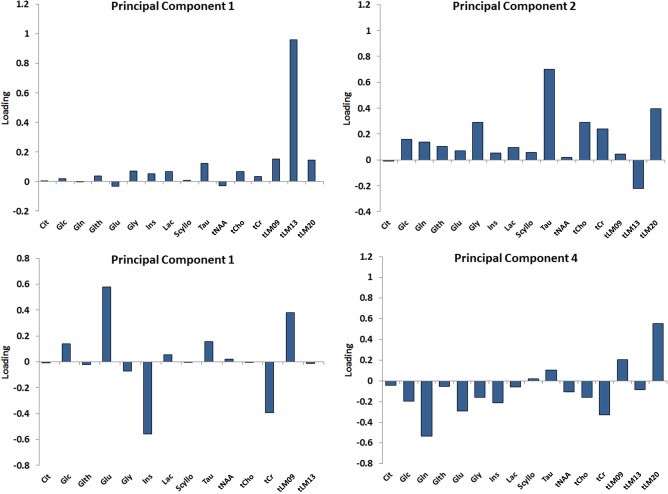
The Bar plot represents the PCA loadings of the metabolite sets for the main principal components demonstrating how the principal components are loaded with the metabolites features.

The bar plot in Figure 3 characterizes PCA loadings of the metabolite set for the four main components. tLM13 has dominated the first principal component. Except Glu and tNAA, all loadings on the first principal component are positive. tLM20, Tau, Gly, tCho, and tCr were highly loaded on the second principal component. For the third principal component, Glc, Glu, mIns, tLM20, and Tau with a negative load were the most dominant. In the fourth principal component, the majority of the metabolites were negatively loaded, however, except Gln, they all were <0.4.

**Figure 4:**
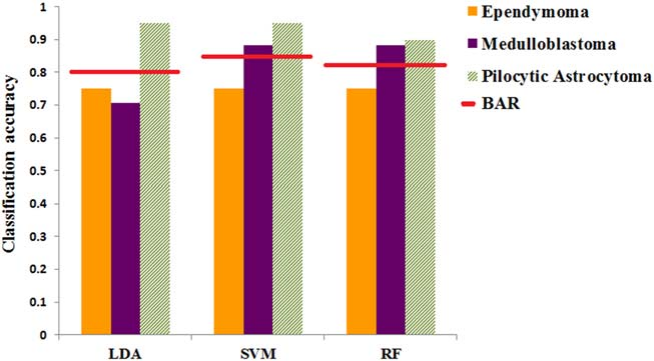
Bar plots represent individual tumor group classification accuracy along with their balanced accuracy comparing performance of the pattern recognition techniques.

**Figure 5:**
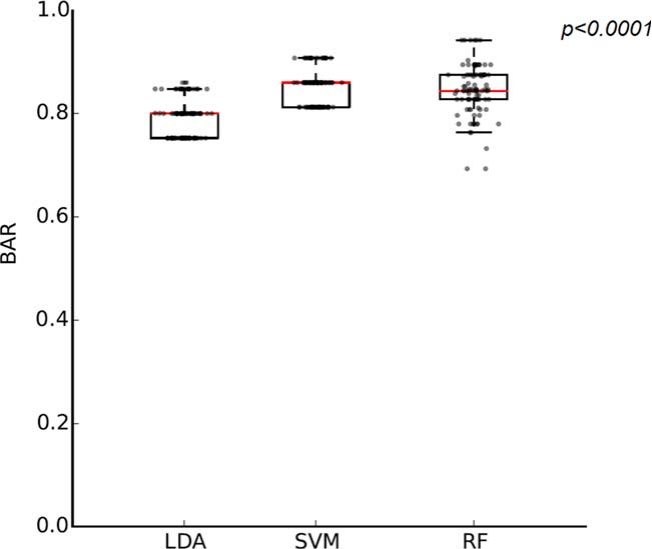
Comparison of the three learning algorithms balanced accuracy rate. Box plot represents the distribution of BAR obtained from 100 runs of oversampling.

BAR of the learning algorithms and their corresponding individual tumor types, F‐measure and G‐mean, are presented in Table 3. SVM (BAR = 0.86) performed favorably in comparison to RF (BAR = 0.84) and LDA (BAR = 0.81) in discriminating between the three tumor types. Figure 4 represents the BAR for the three pattern‐recognition techniques with their accuracy in discriminating individual tumor groups. To further evaluate and describe the differences between the three learning algorithms performance, analysis of variance was carried out on the BAR data obtained from 100 runs of samplings. The three methods showed to have BAR mean of significantly different from each other (*P* < 0.0001) (Fig. 5). RF had higher variance (σ^2^ = 0.002) in comparison to the LDA (σ^2^ = 0.0016) and SVM (σ^2^ = 0.0011).

Comparing methods in view of their performance in classifying EP, all methods had similar accuracy (EP accuracy = 0.75) and misclassified one case as PA. However, EP F‐measure and G‐mean varied amongst the three methods with SVM having the highest values. This finding demonstrates SVM's better ability in balancing the classification performance between the three groups in comparison to the other two methods.

**Table 3:** Balanced Accuracy Rate (BAR) of the Pattern Recognition Techniques along With Individual Tumor Type F‐Measure and G‐Mean.

	BAR	F			G‐Mean		
		EP	MB	PA	EP	MB	PA
LDA	0.81	0.54	0.82	0.9	0.52	0.84	0.91
SVM	0.86	0.85	0.9	0.9	0.86	0.9	0.9
RF	0.84	0.75	0.9	0.87	0.76	0.84	0.87

LDA, linear discriminate analysis; RF, Random forest; SVM, support vector machine; MB, medulloblastoma; PA, pilocytic astrocytoma; EP ependymoma.

For PA, SVM, and LDA achieved the highest accuracy (F‐measure = 0.9, PA accuracy = 0.95). For the MB group, both SVM and RF had highest number of correctly classified cases (F‐measure = 0.9, MB accuracy = 0.88). The misclassification spread across different tumor types, data sites, and scanner types is summarized in Table 4.

**Table 4:** Summary of the Misclassification Spread across Different Tumor Types, Data Sites, and Scanner Types.

Tumor type	Ependymoma	Medulloblastoma	Pilocytic astrocytoma
Misclassified cases (*n*)	1	3–5	1–2
Site of misclassified cases	Alder Hey Children's Hospital (Liverpool)	Birmingham Children's Hospital The Great North Children's Hospital (Newcastle) Alder Hey Children's Hospital (Liverpool)	Birmingham Children's Hospital Alder Hey Children's Hospital (Liverpool) Nottingham Children's Hospital
Scanner manufacturer	Philips	Philips, Siemens	Philips

**Figure 6:**
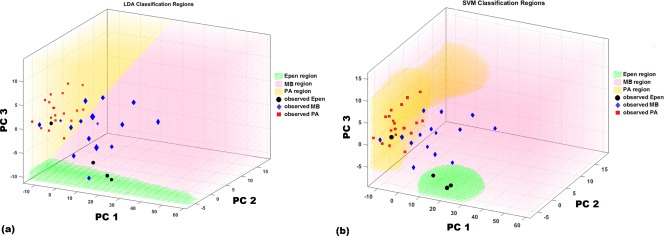
Three‐dimensional scatter plot shows the data clustering for the different tumor types with (**a**) LDA and (**b**) SVM decision boundaries. The graph is oriented so that each clustering can be seen form the best angle with a clear boundary.

The 3D scatter plot in Figure 6 represents LDA and SVM estimated regions. SVM provided improved estimated class boundaries for the three tumor groups that resulted in less overlap between clustered data points and estimated regions from dissimilar tumor types. This difference is more apparent in MB where three cases assigned incorrectly by LDA were correctly classified by SVM.

## DISCUSSION

This is the first multi‐center study investigating 3T MRS and pattern classification for pediatric tumors. Data represented here are from four different centers collected from 3T scanners at short echo time from three common types of childhood tumors.

Mean metabolite concentrations were shown to differ between tumor types with some individual metabolites differing significantly (Mann‐Whitney U test *P* < 0.05) between specific pairs of tumors. The metabolites identified to be significantly different between the three tumor types at 3T in this study generally agrees with previously reported metabolites for the same tumors at 1.5T 6. Exceptions are metabolites such as Glc, Glu, tLM09, and tLM20 that were reported to be significantly different at 1.5T, and Lac that was found to be significantly different at 3T.

For the 3T data set under study, tCho was higher (*P* = 0.01) in MB compared to PA in agreement with tCho as an indicator of cell proliferation and tumor malignancy 18. EP and MB had higher concentrations of lipids and macromolecules compared with PA that is associated with hypoxia, apoptosis, and necrosis and linked to high malignancy and poor survival 3, 19. Tau concentration was significantly higher in MB than PA (*P* = 0.0001) and EP (*P* = 0.001) 20, 21. Similarly mIns concentration was significantly higher in EP than MB and PA (*P* = 0.05).

Glc and Glu were not significantly different between the three tumor types as reported in pervious 1.5T studies, but Lac was significantly different between PA and MB (*P* = 0.05). This finding can be because of difference in sample size or increased resolution at 3T allowing more accurate quantitation.

Applying pattern recognition techniques to the metabolite profile data, a maximum balanced accuracy of 86% was achieved for discriminating between astrocytoma, ependymoma, and medulloblastoma. High classification rates are seen despite the relatively small number of cases used to train the classifiers and the inclusion of data from more than one scanner type. The achieved accuracy was better than the reported BAR obtained from short time echo 1.5T data from ten different international centers where similar tumor types and similar data skewness were studied 6. Using LDA for classification, Vicente et al. 6 reported a BAR of 0.79 for tumors from all regions of the brain. However, the reported 1.5T BAR for LDA is in a very close agreement with LDA accuracy when 3T data is used (3T LDA BAR = 0.81).

The discrimination power of the pattern recognition techniques in classifying the ependymoma group alone was on average 75%. This is mainly because of the imbalanced nature of the data. Although SMOTE was used to overpopulate the ependymoma group by 100% to correct for this skewness, the data distribution still remained imbalanced because of the small number of tumors in this group.

In comparing the performance of different pattern recognition techniques, SVM was a fast, easily trained, and reliable discriminator with the highest balanced accuracy rate. This method, with medium flexibility, avoids under‐ or overfitting of the data. SVMs strongly draw on variation methods in their construction and are designed to yield the best estimate of the optimal separating hyperplane (Fig. 6b). SVM outperforms conventional pattern recognition methods especially when the number of training data is small and number of input variables is large. This is because the conventional pattern recognition methods do not have the mechanism to maximize the margins of class boundaries. Therefore, if some mechanism is introduced to maximize the margins, the generalization ability is improved 22.

Future work should focus on optimizing pattern recognition techniques to classify a wider range of tumor types using 3T MRS. Moreover, a separate prospective study based on an independent test set to ascertain the accuracy of non‐invasive prognostic biomarkers, especially for the minority class, provided by 3T MRS is required.

In conclusion, this study demonstrates the ability and high diagnostic accuracy of 3T MRS in detecting key differences in the metabolite profiles for the main types of childhood tumors. Classification performance of 3T MRS compares favorably with previously reported multi‐center data sets at 1.5T. 3T MRS with automated processing and pattern recognition providing a useful technique for accurate, non‐invasive diagnosis and classification of childhood brain tumors and thereby a powerful diagnostic tool for clinical practice.
